# Histopathology of ameloblastoma of the jaws; some critical observations based on a 40 years single institution experience

**DOI:** 10.4317/medoral.18006

**Published:** 2011-12-06

**Authors:** Doenja Hertog, Elisabeth Bloemena, Irene H A Aartman, Isaäc van-der-Waal

**Affiliations:** 1 Department of Oral and Maxillofacial Surgery/Oral Pathology, VU University Medical Center (VUmc)/ Academic Centre for Dentistry Amsterdam (ACTA), Amsterdam, The Netherlands; 2 Department of Social Dentistry and Behavioural Sciences, Academic Centre for Dentistry Amsterdam (ACTA), Amsterdam, The Netherlands

## Abstract

The aim of the present study is to examine all cases of intraosseous benign ameloblastomas treated between 1970 and 2010 in a single institution and to look for a possible correlation between the histopathological aspects and the demographical and clinical parameters, as well as the treatment outcome. The data of a total number of 44 patients were retrieved from the records. Nine patients were excluded because of doubt about the correct diagnosis (8 patients) or because of an extra-osseous presentation (1 patient). No statistically significant differences were found between the histopathological (sub)types of ameloblastomas and the demographical and clinical parameters, nor between the histopathological (sub)types and treatment outcome. Of the 28 patients treated by enucleation, in 17 patients one or more recurrences occurred, with no significant predilection for any histopathological (sub)type, including the unicystic type. There were no significant differences in the recurrence rate after enucleation in patients below and above the age of 20 years either. In six out of 17 patients with a recurrence, the recurrent lesion showed a different histopathological subtype than was encountered in the primary. In two cases a change from solid/multicystic to desmoplastic ameloblastomas was noticed. In conclusion, the current histopathological classification of benign intraosseous ameloblastoma does not seem to have clinical relevance with the possible exception of the luminal unicystic ameloblastoma that has been removed in toto, unfragmented. Since no primary desmoplastic ameloblastomas were encountered in the present study no further comments can be made on this apparently rare entity.

** Key words:** Odontogenic tumours, ameloblastoma, histopathology.

## Introduction

The ameloblastoma is a histologically almost always benign odontogenic tumour of the jaw bones. However, it has a strong tendency to recur after conservative surgical removal. Extra-osseous occurrence is rather exceptional. Malignant ameloblastomas are extremely rare. The aetiology is unknown. The incidence of ameloblastomas is estimated at 0,5 per million population per year, although in some parts in the world, e.g. South Africa, a higher incidence has been reported (1,2).

Clinically, the tumour often presents as an otherwise asymptomatic swelling of the posterior mandible, frequently being associated with an unerupted tooth. Most patients are aged between 30 and 60 years at the time of diagnosis. There is no gender predilection. Multiple presentation is exceedingly rare. On conventional radiographs the ameloblastoma may present as a unilobular or multilobular corticated radiolucency. Bony septae may result in a honeycomb appearance. Resorption of roots may or may not be present. The radiographic differential diagnosis includes a variety of odontogenic cysts and tumours, particularly the keratocystic odontogenic tumour, as well as nonodontogenic cysts and tumours, such as a central giant cell lesion, fibroosseous lesions and simple bone cyst. It has been mentioned that the desmoplastic ameloblastoma is often characterized radiographically by a mottled, mixed radiolucency/radiopacity with diffuse margins, suggestin a fibroosseous lesion (3).

**Figure 1:**
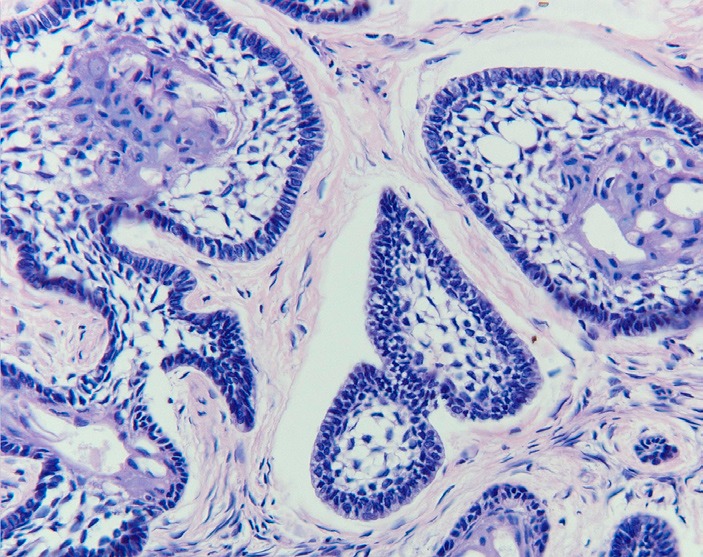
Follicular ameloblastoma showing peripheral palisading and central reticulum stellate pattern (H.E.; orig.magn. x 200).

**Figure 2:**
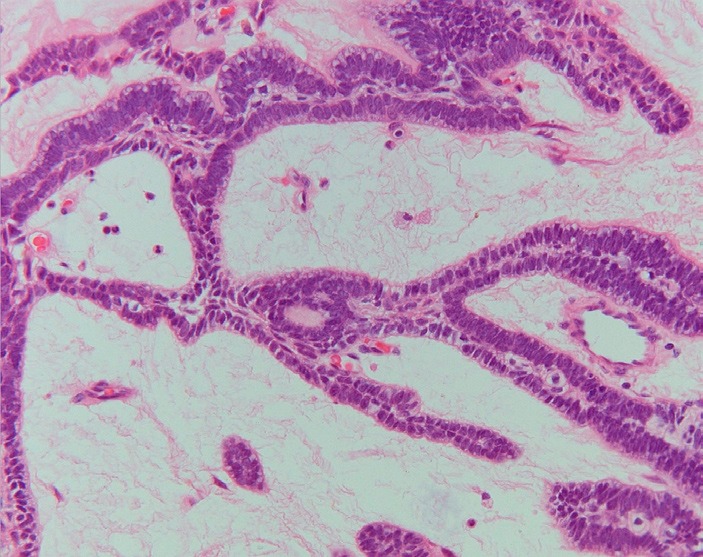
Plexiform ameloblastoma with anastomosing strands and cords of tumour cells (H.E.; orig.magn. x 200).

**Figure 3:**
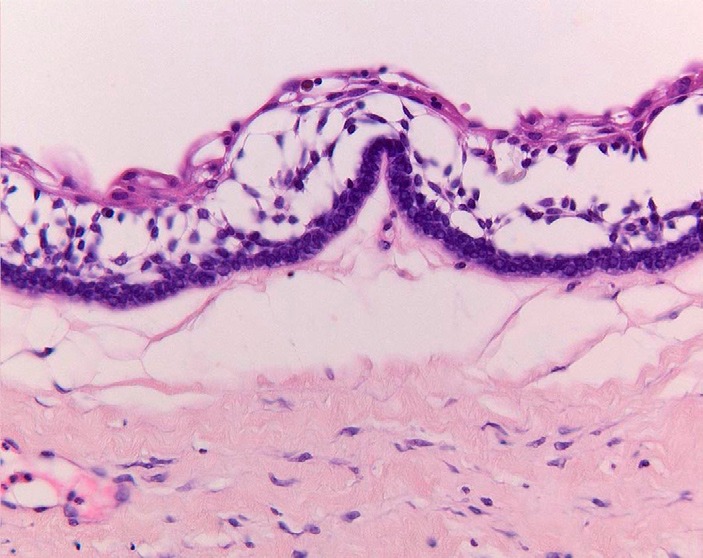
Unicystic ameloblastoma (luminal type), showing ameloblastomatous epithelial lining the "cyst" wall (H.E.; orig. magn. x 200).

**Figure 4:**
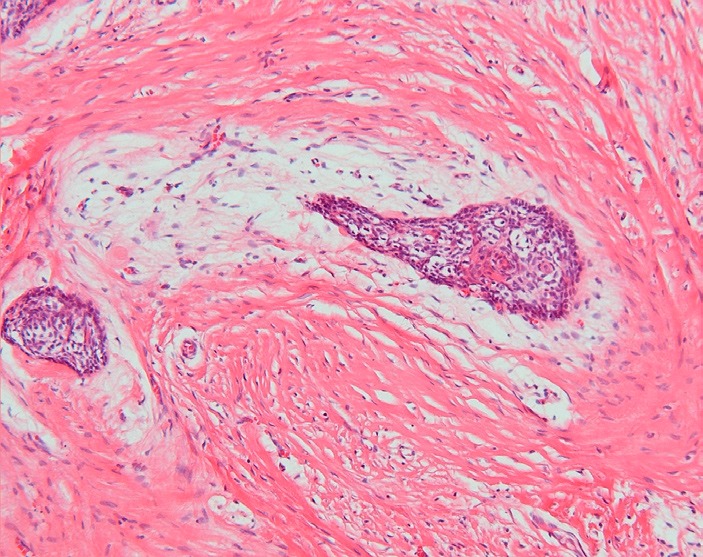
Desmoplastic ameloblastoma. Epithelial tumour islands surrounded by a zone of loose-structured connective tissue (H.E.; orig.magn. x 100).

In the 2005 World Health Organization classification the benign ameloblastoma is divided into 1) solid/multicystic, 2) extra-osseous/peripheral, 3) desmoplastic, and 4) unicystic (3). The solid/multicystic ameloblastoma can histopathologically be divided into a follicular and a plexiform type (Figs. 1 and 2); the follicular type can be further subdivided into a spindle cell type, an acanthomatous type, a granular type and a basal cell type (3). The plexiform type contains basal cells arranged in anastomosing strands with an inconspicuous stellate reticulum. The stroma is usually delicate, often with cystlike degeneration (3). The unicystic ameloblastoma represents an ameloblastoma variant that on gross examination, and not based on the appearance on the radiograph, presents as a cyst. Two histopathological variants are recognized, being the luminal variant and the mural variant (3) (Fig. 3). The extraosseous type shows the histopathogical cell types and patterns as seen in the solid/multicystic type. In the desmoplastic type the stromal component dominates, compressing the odontogenic epithelial components (3) (Fig. 4). 

The preferred treatment of the ameloblastoma is wide surgical removal, with the possible exception of the luminal variant of unicystic ameloblastoma for which enucleation may be justified (4). 

The aim of the present study is to examine all cases of intraosseous benign ameloblastoma registered as such in the period between 1970 and 2010 and to look for a possible correlation between the histopathological aspects and the demographical and clinical parameters as well as the treatment outcome, particularly in patients who initially have been treated by enucleation. Furthermore, the aim is to examine whether histopathological subtypes differ between primary ameloblastomas and one or more of their recurrences. 

## Material and Methods

In the period between September 1970 and September 2010, 44 cases of a benign ameloblastoma and one case of a malignant ameloblastoma were encountered in the files of the Department of Oral and Maxillofacial Surgery/Oral Pathology at the VUmc/ACTA, Amsterdam, The Netherlands. The single case of a primary malignant ameloblastoma has been excluded from the present study. 

Nine of the 44 patients were excluded because of doubt about the correct diagnosis of ameloblastomas (8 patients) or because of an extraosseous presentation (1 patient). Of the remaining 35 patients data on age, gender, localization, radiographs, type of treatment and recurrences were retrieved from the files ( Table 1). Of the 28 patients initially treated by enucleation, 17 patients experienced one or more recurrences, including 11 patients treated previously elsewhere. In one patient a single cervical lymph node metastasis was found in a recurrent, otherwise benign ameloblastoma (“metastatic ameloblastoma”); this patient has been described in more detail elsewhere (5). The mean follow-up of the patients amounted 8.3 years.

The histopathological typing of the biopsies and surgical specimens has been performed by one experienced oral pathologist. The intraobserver variation with regard to the histopathogical subtyping at an interval of six months has been assessed as well. Since it was not possible to characterize all solid/multicystic types in either a follicular or a plexiform type, a category of mixed follicular/plexiform type has been introduced. 

The results were statistically analysed using the Kappa, Student T-test, Chi square test and the Anova test. 

## Results

The results are shown in ( Table 2 and  Table 3). No statistically significant differences were found between unicystic and solid/multicystic ameloblastomas with regard to age and gender (p=0.926 and p=0.735, respectively). Unicystic ameloblastomas only occurred in the mandible (p=0.016) and the solid/multicystic ameloblastomas mainly occurred in the mandible (p=0.004). There was no significant difference in the recurrence rate after enucleation of a unicystic or a solid/mulitcystic ameloblastoma (p=0.544). There were no significant differences in the recurrence rate after enucleation in patients below and above the age of 20 years either.

No significant differences were found between the subtypes of solid/multicystic ameloblastoma with regard to age, gender, localization and treatment outcome ( Table 3).

In 17 of the 28 patients treated with enucleation, one or more recurrences were observed. In six of these patients, the histopathological type of the recurrence differed from the primary tumor. For example, in two patients with an initial plexiform and mixed type ameloblastoma, the recurrence showed a desmoplastic variant. The unicystic ameloblastomas did not recur as a unicystic lesion.

In ( Table 4) the intraobserver variation is shown, the kappa being 0.766. The variation was mainly found in typing follicular and plexiform type, versus mixed type ameloblastoma.

**Table 1:**
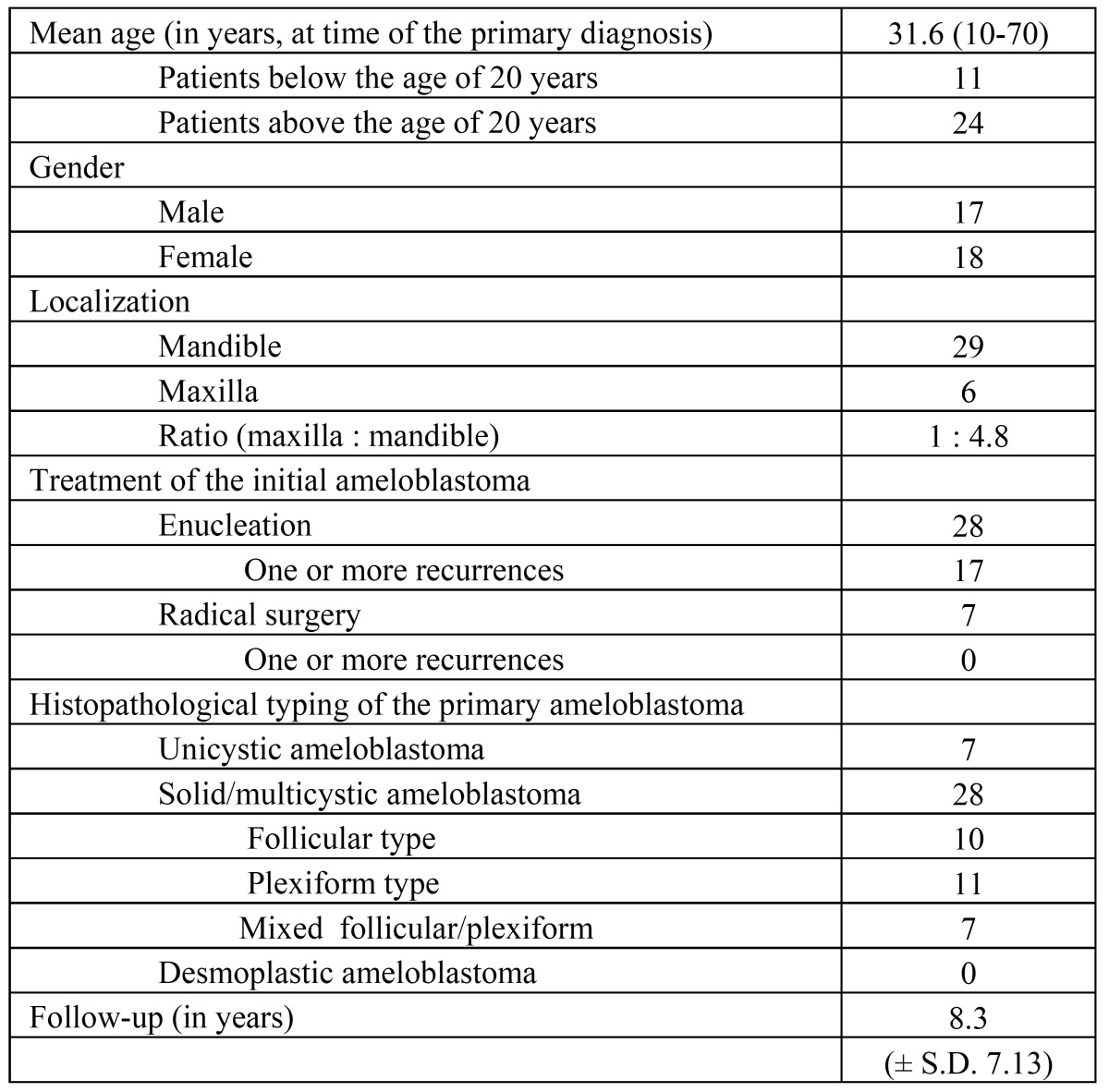
Demographical and clinical data, and histopathological typing of patients with a benign intraosseous ameloblastoma (n=35)

**Table 2:**
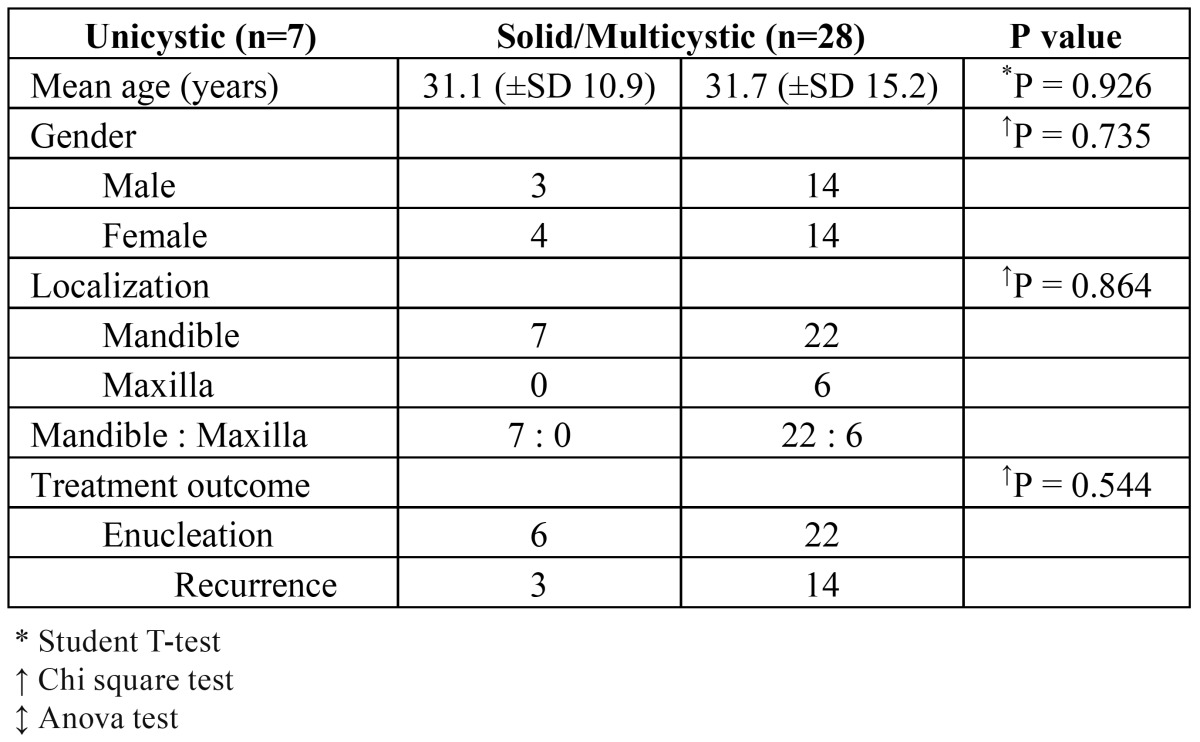
Relation between unicystic versus multicystic types on demographic and clinical parameters (n=35)

**Table 3:**
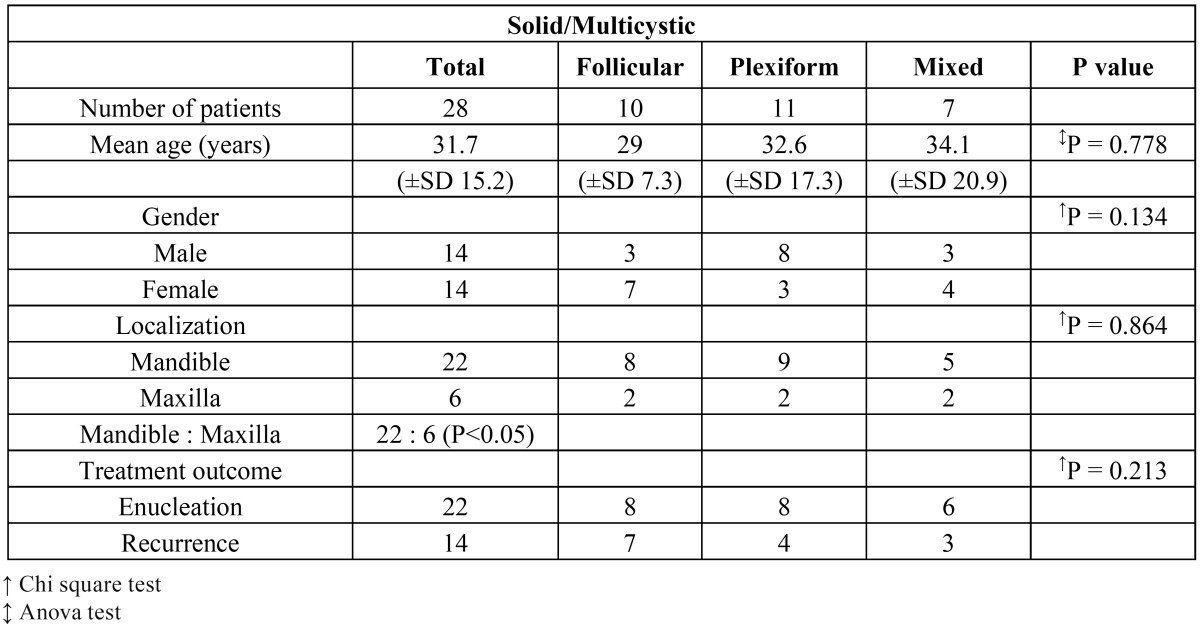
Relation between the three subtypes of solid/multicystic ameloblastomas and demographic and clinical data (n=28)

**Table 4:**
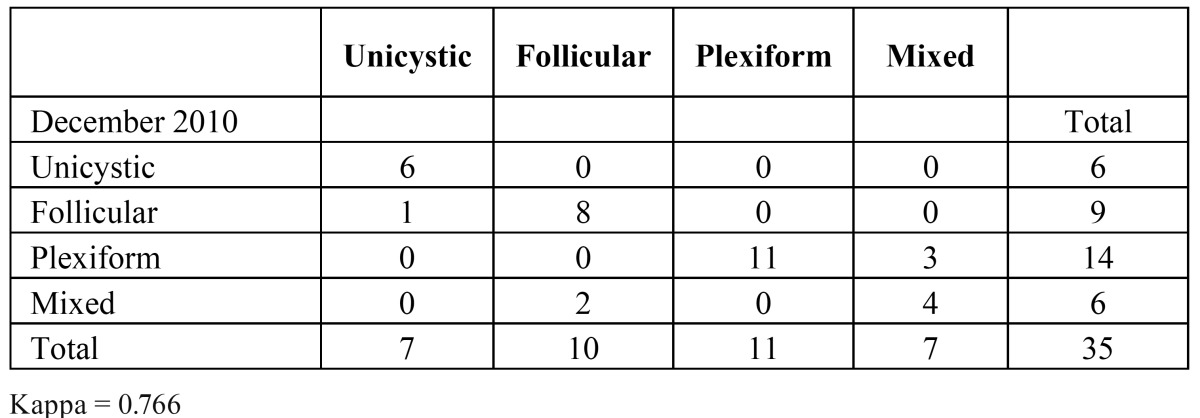
Intraobserver variation in the histopathological subtyping of ameloblastomas (n=35)

## Discussion

 The excluded patients. Nine patients were initially diagnosed with a benign ameloblastoma but have been excluded because of extra-osseous localization (1 patient) or because of doubt about the correct diagnosis of ameloblastoma in the initial lesion (8 patients). In four of these eight patients some nests of ameloblastomalike cells were found in the follicle of a surgically removed wisdom tooth during routine microscopic examination. In such event the question arises how many ameloblastomalike cells are required to justify the diagnosis of ameloblastoma (6). In several studies the presence of ameloblastomalike cells in follicles of asymptomatic third molars has been reported , the percentages varying from 1.5% up to 11% (7-9). In such instances, it seems justified to follow-up such patients for a somewhat arbitrarily chosen period of ten years. 

Another excluded patient was diagnosed with a metastasis of a previously treated cutaneous basal cell carcinoma, extending into the cortical bone of the mandible and mimicking an ameloblastoma. Yet another patient was previously diagnosed with a diagnosis of squamous odontogenic tumour (SOT) (10); the recurrent lesion clearly showed the histological features of an ameloblastoma. One patient with a recurrent ameloblastoma was initially diagnosed as a keratocystic odontogenic tumor (KCOT). The immunohistochemical marker calretinin, which is supposed to discriminate between KCOT and ameloblastoma (11,12), was not helpful in this case One patient was excluded because of a possible diagnosis of odontoameloblastoma. 

 Demographical data. In our series the demographical data were in accordance with the results of other studies. No relation was found with the histopathological subtypes (13,14). Patients from some of the developing countries and dark-skinned patients are younger and Asian patients with an ameloblastoma seem slightly older than Caucasian patients (15). In the present series 11 patients were below the age of 20 years. The age limit of 20 years has been used in the study by Ord et al. (16). There were no significant differences in the recurrence rate after enucleation in patients below and above the age of 20 years. This result does not give support to the belief that ameloblastomas in children behave in a less aggressive way than in adults (16).

It is known that socioeconomic conditions have a major impact on demographic as well as on clinical outcome (17). In the Netherlands most ameloblastomas are found during routine radiographic examination by the dentist. Therefore, one would expect a younger age than in patients diagnosed with an ameloblastoma in some of the developing countries. Apparently, this is not the case (18). There may be genetic and/or external factors influencing the pathogenesis of ameloblastomas that might explain this age discrepancy.

 Clinical data. There were no significant differences between the clinical parameters of the different types of ameloblastoma in comparison with the data from the literature (19). In our series only mandibular unicystic ameloblastomas were observed. The reported prevalence for maxillary localization varies between 8 to 33% in all unicystic cases (18). The prevalence of unicystic ameloblastomas in the present series (7 out of 35 cases, being 20%) is rather high compared to the prevalence figure of 5% of all ameloblastoma cases reported by Darshani Gunawardhana et al. (19). 

 Treatment outcome. The treatment outcome, i.e. recurrence rate after enucleation, was similar for all histopathological (sub)types, including the unicystic types. This is in contrast to the general belief that unicystic ameloblastomas have a lower recurrence rate and, therefore, might be treated less aggressively (18). The recurrence of unicystic ameloblastomas may be explained by fragmental removal with possible tumor spill.

All patients in whom a preoperative diagnosis of ameloblastoma was available were advised to have radical surgery. In the 28 patients who have been treated by enucleation, the recurrence rate in these patients amounted approximately 60 percent (17 out of 28 patients) during a mean follow-up of 8.3 years.

In eight of the 11 patients below the age of 20 years (73%), a recurrence was observed. A lower recurrence rate in children as being reported in other studies might be explained by a higher percentage of unicystic ameloblastomas in those studies (16). 

 Histopathogical aspects. In nine cases of solid/multicystic ameloblastoma an equal distribution of a follicular and a plexiform pattern was noticed within one sample. For these cases a category of mixed type follicular and plexiform was added. In five of these nine cases there was a rather high intraobserver variability, the overall intraobserver kappa being 0.766. The variation was mainly found in typing follicular and plexiform type, versus mixed type ameloblastoma.

In six of 17 patients with a recurrence, the primary lesion was typed different. In two cases a change from solid/multicystic ameloblastoma to desmoplastic ameloblastomas was noticed. It is tempting to speculate that the desmoplastic type mainly or perhaps even exclusively occurs in cases of recurrence. However, by examining the stromal reaction for collagen type VI in desmoplastic and solid/multicystic stroma, it was concluded that the desmoplastic stroma was not to be interpreted as simple scar tissue but as newly produced connective tissue; it has been suggested that TGF-beta produced by epithelial tumour cells of desmoplastic ameloblastoma play a part in the prominent desmoplastic matrix formation (20,21). Also in view of the clinicoradiographic features of desmoplastic ameloblastomas, its recognition as a separate entity seems fully justified (3,22).

The recurrence of three unicystic ameloblastomas can probably be explained by fragmentation during removal of the primary tumor. It is, indeed, often difficult to remove a cystic lesion in tot without fragmentation. It should be realised that a diagnosis of unicystic amoloblastoma may not always be easy to establish with certainty (23). Parts of the epithelial lining of a (uni)cystic ameloblastoma may lack the pathognomonic features an ameloblastoma. A biopsy of a primary unicystic ameloblastoma or a biopsy of a recurrent, solid/multicystic ameloblastoma, may not always show the typical features of ameloblastoma, which may result in an underdiagnosis and, as a result, possibly in incorrect management.

In the 11 patients below the age of 20 years, nine solid/multicystic and two unicystic ameloblastomas were noticed. This is in contrast to the suggestion that unicystic ameloblastomas are more common among children than among adults (16). 
